# Proportion and Correlates of Psychiatric Morbidity Among Psychiatry-Assessed Oncology Inpatients

**DOI:** 10.3390/diseases13110350

**Published:** 2025-10-24

**Authors:** Ana-Maria Paslaru, Iulian Bounegru, Catalin Plesea-Condratovici, Moroianu Marius, Anamaria Ciubară

**Affiliations:** 1 Doctoral School of Biomedical Sciences, “Dunărea de Jos” University, 800008 Galați, Romania; annapaslaru@gmail.com; 2 Medical Department, Faculty of Medicine and Pharmacy, “Dunărea de Jos” University, 800008 Galați, Romania; anamburlea@yahoo.com; 3 Competences Centre, Interfaces-Tribocorrosion-Electrochemical Systems, “Dunărea de Jos” University, 800008 Galați, Romania; 4 Morphological and Functional Sciences Department, Faculty of Medicine and Pharmacy, “Dunărea de Jos” University, 800008 Galați, Romania; 5 Department of Dental Medicine, Faculty of Medicine and Pharmacy, “Dunărea de Jos” University, 800008 Galați, Romania; moroianu.g.marius@gmail.com

**Keywords:** oncology, psychiatric morbidity, anxiety, depression, urogenital cancers, Hospital Anxiety and Depression Scale, ECOG

## Abstract

**Background/Objectives:** Psychiatric morbidity is frequent in oncology, yet prevalence and correlates differ across tumour sites. Urogenital cancers, in particular, involve psychosocial stressors related to sexuality, fertility, continence, and body image, which may intensify anxiety and depression. This study aimed to estimate the proportion of psychiatric morbidity among psychiatry-assessed oncology inpatients in a real-world hospital setting to compare urogenital with non-urogenital malignancies and to examine clinical correlates and hospitalisation outcomes. **Methods:** We conducted a retrospective analysis of 174 oncology inpatients who were evaluated by liaison psychiatry and completed the Hospital Anxiety and Depression Scale (HADS) during admission to a tertiary hospital in Galați, Romania, between 2019 and 2022. All patients completed the Hospital Anxiety and Depression Scale (HADS) and underwent liaison psychiatry evaluation. Mixed anxiety–depressive disorder (ICD-10 F41.2) was the primary psychiatric outcome. Demographic, clinical, and functional data—including Eastern Cooperative Oncology Group (ECOG) performance status—were extracted from medical records. Comparative and multivariable analyses were performed to identify predictors of severe depressive symptoms (primary outcome, HADS-D ≥ 11) and to explore associations with length of stay and costs. **Results:** Overall, 59% of patients had elevated HADS-Anxiety and 62% elevated HADS-Depression, while 40% received a psychiatric diagnosis. Mixed anxiety–depressive disorder predominated, especially in cervical (95%), bladder (100%), and prostate (≈70–75%) cancers. Urogenital cancers showed significantly higher rates of anxiety/depression than non-urogenital cancers (85% vs. 46%, *p* < 0.01). Poorer ECOG status independently predicted severe depressive symptoms (OR 3.6, 95% CI 2.1–6.2, *p* < 0.001). Psychiatric morbidity was associated with a trend toward longer LOS (median 12 vs. 9 days, *p* ≈ 0.08) and ≈10% higher hospital costs. **Conclusions:** Anxiety and depression were highly frequent among psychiatry-assessed oncology inpatients, particularly in urogenital malignancies. Functional impairment strongly correlates with psychiatric morbidity. These findings underscore the need for systematic screening and risk-stratified psycho-oncologic interventions to improve patient outcomes and resource utilisation.

## 1. Introduction

Psychiatric morbidity is common across the cancer trajectory, with anxiety and depressive disorders occurring more often in oncology than in the general population. Contemporary clinical practice guidelines recommend routine case-finding and stepped, integrated psycho-oncology to mitigate impacts on adherence, quality of life, and clinical outcomes [1,2].

Recent syntheses indicate that symptom burden remains significant across various settings and regions. For instance, pooled data from recent comprehensive reviews show anxiety and depression prevalences around or exceeding 20% in mixed cancer populations, with notable variation by geography and methodology; some regions exhibit even higher rates. These patterns highlight the importance of systematic screening and prompt referral within comprehensive oncology services [3,4,5].

In psycho-oncology, distress denotes a multidimensional spectrum of emotional suffering that can interfere with coping and care; within this spectrum, anxiety and depressive symptoms are its most quantifiable core components in routine services. Accordingly, contemporary guidelines and syntheses commonly operationalise clinically significant distress via validated affective measures, with the Hospital Anxiety and Depression Scale (HADS) used as a pragmatic proxy in medically ill populations. This approach minimises somatic overlap with cancer/treatment effects and enables stepped, guideline-concordant care when thresholds are exceeded [1,2,6].

Tumour site-specific stressors can further shape psychological burden. Urogenital malignancies involve concerns about sexual function, continence, fertility, and body image that may amplify distress relative to some other cancers. In bladder cancer, sexual health remains an unmet supportive care domain after treatment; in cervical cancer, clinically significant anxiety/depression is frequently observed; and in prostate cancer, androgen-deprivation therapy (ADT) has been associated with increased risk of depression, underscoring the need for proactive monitoring in this group. These gradients are consistent with integrative frameworks linking cancer-related biological stress (e.g., inflammation), treatment-related effects (e.g., ADT), and affective morbidity in oncology, as highlighted in recent syntheses and related regional work [7,8,9,10,11]

Validated patient-reported measures facilitate the efficient detection of distress. The Hospital Anxiety and Depression Scale (HADS) is widely used in oncology; foundational psychometric and normative work, complemented by cancer-specific applications in diverse settings, supports its use for screening anxiety (HADS-A) and depression (HADS-D) in medically ill populations [12,13]. Importantly for non-specialists, the HADS was designed to prioritise cognitive–affective content (e.g., anhedonia, worry, tension) and exclude somatic items that are liable to be confounded by cancer symptoms (e.g., fatigue, insomnia), thereby capturing the affective core of distress. Elevated HADS scores are therefore routinely treated as operational markers of clinically significant distress that warrant stepped psycho-oncologic response, consistent with international guidance [1,2,6].

Clinical and demographic correlates modulate risk. Across inpatient and perioperative samples, worse functional status—indexed by the Eastern Cooperative Oncology Group (ECOG) performance scale—tends to co-occur with higher anxiety/depression symptomatology [14,15]. Recognising such correlates enables risk-stratified pathways aligned with guideline recommendations [1,2].

Beyond symptom prevalence, distress has practical implications for care delivery and resource use. In general hospital settings, early and proactive consultation-liaison psychiatry is associated with reduced length of stay in randomised and service evaluation studies, suggesting that timely identification and management of psychological problems can improve patient flow and outcomes [16,17].

Against this backdrop, we conducted a retrospective analysis of 174 oncology inpatients at a single tertiary centre. All patients underwent standardised HADS screening, with consultation-liaison psychiatry involvement when indicated. We aimed to (i) quantify the proportion of psychiatric morbidity among oncology inpatients assessed by liaison psychiatry in a real-world hospital setting; (ii) compare psychological burden between urogenital (cervical, prostate, bladder) and non-urogenital (lung, head and neck, breast, rectal) malignancies; (iii) examine demographic and clinical correlates, including ECOG performance status; and (iv) explore associations with utilisation-relevant outcomes such as hospital length of stay and treatment interruptions. The objective is to inform risk-stratified, guideline-concordant psycho-oncologic care focused on tumour site- and function-informed needs, while making explicit that distress is operationalised via HADS-A/D thresholds in this inpatient, liaison psychiatry-assessed cohort [1,2,6,16,17].

## 2. Methods

### 2.1. Study Design and Participants

This study was a retrospective observational analysis of cancer in patients treated over four years (January 2019–December 2022) at the County Emergency Clinical Hospital “Sf. Ap. Andrei” in Galaţi, Romania. Only oncology inpatients who were referred to and evaluated by the hospital’s liaison psychiatry service were included. This subgroup represents patients in whom psychological assessment was indicated during routine care, rather than the entire oncology inpatient population. During the study period (2019–2022), approximately 1240 oncology inpatient admissions were recorded in the tertiary centre. Among these, 310 patients (25%) completed routine psychological screening with the Hospital Anxiety and Depression Scale (HADS) as part of standard care. Of the screened patients, 186 (15% of total admissions) were referred to liaison psychiatry for evaluation, and 174 met all inclusion criteria and were retained for analysis after excluding those with incomplete data or pre-existing major psychiatric disorders. This flow summarises the patient pathway from admission to inclusion and indicates that the study represents the subset of oncology inpatients both screened and psychiatry-assessed. All data were collected from existing medical records without any intervention in patient care, allowing for real-world clinical observation without affecting treatment. The study population included 174 adult oncology inpatients (≥18 years) who were evaluated by the liaison psychiatry team during hospitalisation and completed the Hospital Anxiety and Depression Scale (HADS). These patients formed the psychiatry-assessed subgroup within the larger oncology service [18]. Inclusion required completion of the Hospital Anxiety and Depression Scale (HADS) during admission and the availability of an Eastern Cooperative Oncology Group (ECOG) performance status score in the records. Only patients with complete medical records and all necessary data (demographics and clinical and psychiatric assessments) were included. Exclusion criteria included any documented prior psychiatric disorders (e.g., major depression, psychotic or personality disorders), any other primary psychiatric diagnosis during the index admission (such as ICD-10 codes F32 for major depressive episode or F41.1 for generalised anxiety), inability to cooperate with psychiatric evaluation (e.g., acute confusion or severe neurological comorbidities), or missing HADS/ECOG data. Following these criteria, a final sample of 174 patients was assembled. For subgroup analyses, patients were categorised by cancer type into a urogenital cancer group (n ≈ 73)—including malignancies of the prostate, cervix, or urinary bladder—and a non-urogenital cancer group (n ≈ 101) comprising other solid tumours (such as head–neck, breast, lung, and colorectal cancers). This grouping was used to compare psychiatric outcomes between urogenital and other cancer patients.

### 2.2. Clinical Data Collection

Trained researchers extracted clinical and demographic data from the hospital’s electronic records using a standardised form. Patient demographics included age, sex, and residence (classified as urban or rural). Oncologic variables included the primary cancer site (tumour location), which was later grouped for analysis as noted above. Functional status at admission was recorded using the ECOG Performance Status scale (ranging from 0 = fully active to 4 = completely disabled). We also recorded the dates of hospital admission and discharge to calculate the length of stay (in days) for each patient. Discharge status noted the patient’s condition at discharge (e.g., improved, stable, or deceased). Additionally, we collected information on whether the patient’s oncology treatment was interrupted or prematurely terminated due to their psychiatric condition or personal request (as an indicator of non-compliance). Specifically, any documented instances of patients refusing or delaying recommended cancer therapy, or leaving the hospital against medical advice, were recorded as treatment interruptions when documented in the charts. Each patient’s final oncologic diagnosis and stage (when documented) were obtained from the discharge summary (“diagnostic liber”), and these narrative diagnoses were used to verify cancer type and confirm eligibility criteria (all diagnoses were solid tumours confirmed via pathology or imaging).

### 2.3. Psychiatric Assessment

All included patients received a consultation-liaison psychiatry evaluation during their oncology admission. As part of this assessment, the Hospital Anxiety and Depression Scale (HADS) was administered to quantify symptoms of anxiety and depression. HADS is a validated 14-item self-report instrument with two subscales: 7 items for anxiety (HADS-A) and 7 for depression (HADS-D). Each item is scored 0–3, yielding subscale totals from 0 to 21 for anxiety and depression, respectively [19]. Following standard conventions, we interpreted HADS scores of 8 or above as indicating possible clinically significant anxiety or depression, and scores of 11 or above as denoting probable (definite) cases on each subscale [19,20]. For psychiatric diagnosis, the liaison psychiatrist applied ICD-10 diagnostic criteria. In every case included, the psychiatrist documented a secondary diagnosis of mixed anxiety and depressive disorder (ICD-10 F41.2) in the medical record, meaning both anxiety and depressive symptoms were present in the patient without one clearly predominating and without meeting full criteria for a major depressive or specific anxiety disorder. Where applicable, the psychiatrist also recorded other relevant psychiatric or psychosocial observations. We noted any use of psychotropic medications initiated during the hospital stay as part of the liaison intervention (e.g., anxiolytics or antidepressants), although detailed treatment of the psychiatric condition was not the focus of this study.

### 2.4. Economic Impact and Treatment Adherence Measures

To evaluate the economic impact of comorbid psychiatric morbidity, we collected data on each patient’s hospitalisation costs. This included the direct treatment cost of the oncology admission (in Romanian Lei, as calculated by the hospital’s accounting based on procedures and length of stay) and the corresponding reimbursed amount by the National Health Insurance, which indicates how much of the cost was covered by the insurer [21]. These figures allowed us to estimate the financial burden per patient and any potential gaps between cost and reimbursement. We also examined treatment adherence indicators: if a patient’s anxious–depressive symptoms were noted to affect their compliance (for example, refusal of chemotherapy sessions, delays in treatment, or early self-discharge), this was recorded. Although formal quantification of adherence was limited by retrospective chart documentation, any mention of non-compliance with medical recommendations or premature termination of cancer treatment was treated as a qualitative indicator of treatment interruption. This information was used to assess whether psychiatric symptoms might correlate with poorer oncology treatment completion rates.

### 2.5. Statistical Analysis

Statistical analyses were performed using IBM SPSS Statistics 26.0 (IBM Corp., Armonk, NY, USA). Continuous variables were summarised as mean ± SD or median [IQR], and categorical variables as counts and percentages.

The primary outcome was severe depressive symptomatology, defined as HADS-Depression (HADS-D) ≥ 11. This threshold was prespecified for logistic regression analyses to identify independent predictors of clinically significant depression. Secondary, exploratory outcomes included hospital length of stay (LOS, days) and direct treatment cost (RON).

Bivariate comparisons used independent-samples t, Mann–Whitney U, and χ^2^/Fisher’s exact tests, as appropriate. Variables associated with the primary outcome at *p* < 0.10 in univariate testing were entered into multivariable logistic regression. Assumption checks included assessment of multicollinearity (Variance Inflation Factor < 2.5) and linearity in the logit for continuous predictors and absence of influential outliers (Cook’s distance < 1.0). Goodness-of-fit was verified using the likelihood-ratio test and McFadden’s pseudo-R^2^.

For LOS and cost outcomes, ordinary least-squares (OLS) linear regression was applied after inspecting residual plots for normality, linearity, and homoscedasticity. Log-transformation was used for cost data to normalise distribution. Model fit was assessed using R^2^ and overall F-test *p*-values.

Missing data were minimal (<5% for LOS and cost) and handled by complete-case analysis, as sensitivity testing with mean imputation produced similar estimates. Statistical significance was set at two-tailed *p* < 0.05, and all effect estimates are presented with 95% confidence intervals (CIs).

**Figure 1:**
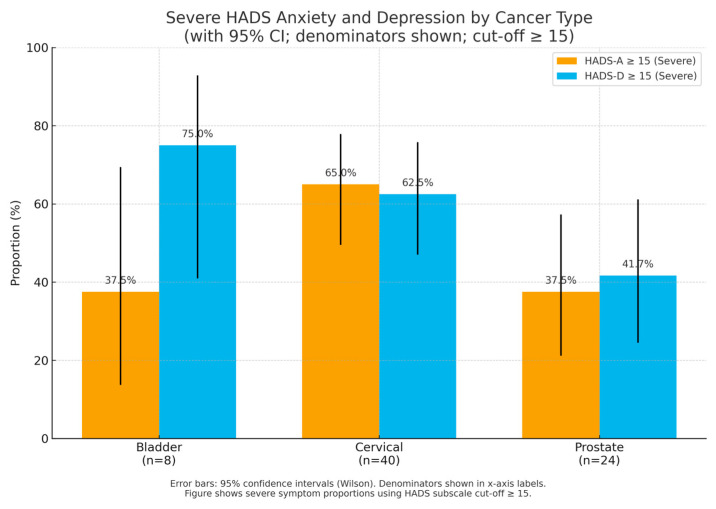
Severe HADS anxiety and depression by cancer type. Bars show proportions (%) among psychiatry-assessed inpatients; denominators are indicated below each tumour type (n). Error bars are 95% confidence intervals (Wilson). HADS = Hospital Anxiety and Depression Scale. HADS = Hospital Anxiety and Depression Scale.

In addition to the primary threshold (HADS-D ≥ 11), an exploratory logistic model was also tested using a more stringent cut-off (HADS-D ≥ 15) to visualise high-severity cases (Figure 1).

### 2.6. Ethical Considerations

The study protocol was approved by the Ethics Committee of the “Saint Apostle Andrew” County Emergency Clinical Hospital, Galați, Romania (approval no. 13682/23 June 2023). Given the retrospective design and exclusive use of anonymised patient data extracted from the hospital information system, the Ethics Committee granted a waiver of informed consent. The study complied with the Declaration of Helsinki and all applicable national regulations on patient data confidentiality.

## 3. Results

### 3.1. Patient Characteristics

#### 3.1.1. Sample Description

A total of 174 patients were included. The median age was 68 years (range, 40–91), with a mean ± SD of approximately 67.0 ± 9.5 years, reflecting a predominantly middle-aged to older oncology population. Women represented 51.1% of the sample (n = 89) and men 48.9% (n = 85). As expected, women were concentrated in the breast and cervical cancer groups, whereas men predominated in the lung, head and neck, and prostate groups. Most patients resided in urban areas (77.0%) versus 23.0% from rural settings, consistent with the hospital’s largely urban catchment and referral patterns.

#### 3.1.2. Cancer Type Distribution

The cohort encompassed seven primary tumour sites (Figure 1). The largest subgroups were head and neck (ORL) cancers (24.1%) and cervical cancer (23.0%), followed by breast (17.2%), prostate (14.4%), colorectal/rectal (10.3%), bronchopulmonary (lung) (6.3%), and bladder (4.6%). Overall, 42% of patients had urogenital malignancies (cervix, prostate, bladder; n = 73) and 58% had non-urogenital cancers (lung, head and neck, breast, rectal; n = 101). The urogenital subset comprised all 40 cervical (female), 25 prostate (male), and 8 bladder (mixed-sex) cases; Table 1 details age, sex distribution, and ECOG performance status by cancer type.

#### 3.1.3. Clinical Status

At admission, performance status was generally poor, with 54.6% of patients having an ECOG score of 3 and 32.2% having an ECOG score of 2, while smaller proportions had ECOG scores of 1 (10.3%) and 4 (2.9%) (Table 1). This distribution indicates substantial functional impairment consistent with advanced disease and/or intensive oncologic therapy. Discharge outcomes mirrored this clinical acuity: “cured” status was rare (1.15%), while 55.8% were discharged improved, 42.0% were stationary, and 1.15% died in-hospital.

The median length of stay (LOS) across the cohort was ~10–11 days (overall range 0–56 days; 0 denotes same-day discharge). The mean LOS was ~11.3 ± 10.0 days. By tumour site, head and neck and cervical cancer patients tended to have longer median stays (~10 days), whereas breast cancer patients had shorter median stays (~6–7 days). In a simple comparison of grouped sites, urogenital vs. non-urogenital cancers showed no significant difference in median LOS (~10 vs. 11 days, *p* > 0.10). Within the urogenital category, bladder cancer patients tended to have longer hospitalisation (median 13.5 days), consistent with the high clinical burden observed in this subgroup. Table 1 provides the full quantitative summary of demographic, tumour site, ECOG, and LOS distributions.

### 3.2. Prevalence of Psychiatric Morbidity

Psychiatric symptom screening and diagnostic coding indicated a substantial burden of psychological comorbidity in this cohort. All estimates refer to the proportion of psychiatric morbidity among oncology inpatients assessed by liaison psychiatry, not among all admitted oncology cases. Based on HADS evaluations, approximately 60% of patients (~104/174) had at least borderline or clinically elevated anxiety and/or depression (HADS subscale ≥ 8). Using the stricter threshold of ≥11 on either HADS subscale, 45% of patients exhibited definite anxiety and/or depression, warranting intervention. In the medical records, ~40% (~70/174) received at least one formal psychiatric diagnosis during oncologic care. The most frequent was mixed anxiety–depressive disorder (ICD-10 F41.2), characterised by concurrent anxiety and depressive symptoms. Additional diagnoses included depressive episode/major depression (≈10%), adjustment disorder with mixed anxiety and depressed mood (≈8%), and isolated anxiety disorders (≈5%, e.g., generalised anxiety). A small fraction (≈3%) had organic mood syndromes or delirium related to medical illness; these were excluded from the primary psychiatric prevalence analysis.

By cancer type, cervical, bladder, and prostate cancers showed the highest rates of mixed anxiety–depressive presentations. Cervical cancer patients exhibited the greatest psychological morbidity: 95% (38/40) met criteria for mixed anxiety–depression or had HADS scores indicating significant distress. Bladder cancer patients also showed a striking rate, with 100% (8/8) meeting criteria for clinically relevant anxiety/depression (predominantly F41.2). Prostate cancer patients had a slightly lower but still substantial prevalence (~70–75%). Among non-urogenital cancers, prevalence was lower overall: breast cancer ~50–60% (often adjustment-related), head and neck (ORL) ~40%, lung ~45%, and rectal ~50% with elevated distress.

These patterns are summarised in Table 2, which reports, by tumour type, the proportion with HADS-Anxiety ≥8, HADS-Depression ≥8, and any psychiatric diagnosis. In brief, cervical and bladder cancers top the list, whereas lung and head and neck cancers have the lowest proportions of diagnosed psychiatric comorbidity (still ≈one-third affected). Collapsing sites into urogenital vs. non-urogenital groups showed a statistically significant difference in distress prevalence (~85% vs. ~45%, χ^2^ test, *p* < 0.01), supporting Hypothesis 2.

Group comparison (urogenital vs. non-urogenital): χ^2^ tests indicate significantly higher prevalence in the urogenital group for HADS-A ≥ 8 and HADS-D ≥ 8 and for any psychiatric diagnosis (all *p* < 0.01).

Mean HADS-total scores by cancer type (reported in text) mirror these differences (e.g., cervical 16.1 ± 2.8; bladder 15.9 ± 2.2; and prostate 14.9 ± 3.8 vs. ~11–13 in other groups), and one-way ANOVA confirmed a cancer type effect on HADS (*p* < 0.05).

Mean psychometric scores mirrored these differences. The mean HADS-total was 16.1 ± 2.8 in cervical cancer, 15.9 ± 2.2 in bladder cancer, and 14.9 ± 3.8 in prostate cancer, versus ~11–13 in other groups. ANOVA indicated a significant effect of cancer type on HADS (F-test, *p* < 0.05); post hoc comparisons highlighted cervical vs. breast and cervical vs. head and neck differences (cervical higher). These findings support that those urogenital cancers—especially cervical—are associated with more severe psychological distress.

### 3.3. Factors Associated with Psychological Distress

**Sex**. Overall, female patients had a higher incidence of anxiety/depression than male patients (52% vs. 38% meeting HADS/diagnostic criteria; χ^2^, *p* = 0.07). Although this trend did not reach strict statistical significance in the full cohort—likely due to confounding by cancer type (many females had breast/cervical cancers with higher distress; many males had ORL/prostate with variable distress)—a stratified comparison within mixed-sex tumour groups (e.g., head and neck and rectal) showed significantly higher mean HADS-Anxiety in females (by ~3 points; *p* < 0.05), suggesting a gender-related vulnerability independent of site.

**Age**. Contrary to expectation, younger (<65) patients did not have a significantly higher prevalence of psychiatric morbidity than older (≥65) patients. Distress was high across age brackets. The correlation between age and anxiety was weak and non-significant (Pearson r = −0.10 with HADS-Anxiety, *p* = 0.20), indicating at most a slight trend toward higher anxiety in younger patients.

**Residence (Urban vs. Rural).** There was no significant difference in distress by residence. Psychiatric diagnosis rates were ~41% urban vs. ~35% rural (*p* = 0.49), and mean HADS scores were virtually identical. This suggests similar in-hospital access to psycho-oncologic resources across residence groups, although the rural sample (n = 40) limits the power to detect small effects.

**Performance Status (ECOG).** As hypothesised, worse ECOG was strongly associated with greater distress. Patients with ECOG 3–4 had ~3.5-fold higher odds of psychiatric diagnosis compared with ECOG 1–2 (logistic regression, OR ≈ 3.5, 95% CI ~1.5–8.0, *p* < 0.01). Mean HADS-Depression was ~3 points higher in ECOG 3–4 than in ECOG 1–2 (10 vs. 7, *p* = 0.004). In practical terms, >60% of ECOG 3–4 patients exhibited significant depressive symptoms vs. ~30% of ECOG 1–2, underscoring the need to prioritise psychosocial support when functional status declines.

**Cancer Type (multivariable).** Beyond the urogenital vs. non-urogenital contrast, multivariable modelling (adjusting for age and ECOG) indicated cervical cancer as an independent predictor of high distress: being a cervical cancer patient conferred ~5-fold higher odds of severe anxiety/depression relative to breast cancer (reference), *p* < 0.05. No other sites remained significant after adjustment, with a non-significant trend toward lower odds in head and neck cancers. These results suggest that specific tumour biology/psychosocial context (not only demographics or performance status) contributes to the elevated distress observed, particularly in cervical cancer.

### 3.4. Impact of Psychiatric Comorbidity on Hospitalisation Outcomes

**Length of stay (LOS).** Patients with a documented anxiety/depression diagnosis or very high HADS scores tended to have longer hospitalisations, although the difference did not reach conventional statistical significance. The median LOS was 12 days in the psychiatric comorbidity group versus 9 days in those without, with a Mann–Whitney U test *p* ≈ 0.08, indicating a trend-level effect rather than a definitive difference. A small number of extreme outliers (stays > 40 days), all with severe medical complications and most with concurrent depressive symptoms, widened the between-group gap; excluding outliers narrowed the mean LOS difference further, reinforcing that any effect of psychiatric comorbidity on LOS in this sample is modest at best.

**Treatment interruptions.** We identified 15 patients (8.6%) who experienced unplanned interruption or premature cessation of cancer therapy (predominantly radiotherapy breaks beyond planned schedules). Within this subset, 73% exhibited significant depressive symptoms. In exploratory logistic regression, severe depression (HADS-D ≥ 15) was associated with higher odds of treatment interruption (OR ~1.8), but this finding was not statistically significant (*p* ~ 0.25), reflecting the limited event count and correspondingly wide confidence intervals. Chart reviews indicated mixed reasons for interruption (medical complications, withdrawal of consent, and—qualitatively—features consistent with apathy/hopelessness), suggesting a clinically relevant but underpowered signal that depressive symptomatology may undermine treatment continuity.

**Healthcare costs.** Although a full economic evaluation was beyond scope, crude comparisons of direct treatment costs per hospitalisation showed ~10% higher mean costs in patients with psychiatric comorbidity, roughly paralleling the longer LOS. We did not perform formal hypothesis testing on costs in this analysis; therefore, this observation should be considered hypothesis-generating and consistent with the notion that psychological morbidity may increase resource utilisation (e.g., extended monitoring, supportive care).

**Figure 2:**
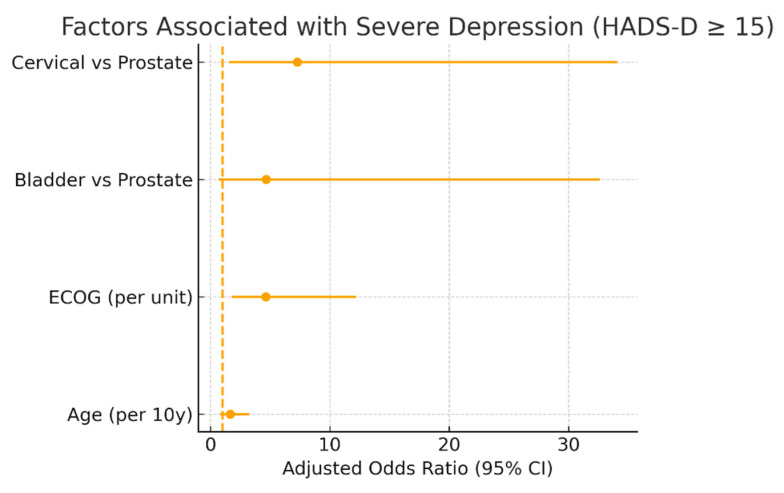
Factors associated with severe depressive symptoms (exploratory analysis, HADS-D ≥ 15). Adjusted odds ratios (95% confidence intervals) from logistic regression model including ECOG performance status, age (per 10 years), and cancer type (reference = prostate). Vertical dashed line indicates OR = 1. Error bars denote 95% CI. Analysis is exploratory and complementary to primary model using HADS-D ≥ 11.

An exploratory model using the higher HADS-D ≥ 15 threshold is shown in Figure 2, illustrating the same direction of associations observed with the primary cut-off (HADS-D ≥ 11). This stricter threshold highlights the robustness of the relationship between poorer functional status (ECOG) and depressive symptom severity, although confidence intervals are wider due to smaller subgroup sizes.

These findings are directionally consistent with our hypotheses: psychiatric morbidity was common (H1) and more prevalent in urogenital cancers (H2), and it tracked with poorer functional status (ECOG) (H3). Regarding hospitalisation outcomes and care continuity (H4), the data suggest trend-level associations—longer LOS and more interruptions among those with severe depression—but do not establish statistical significance in this sample. Larger, prospectively powered studies are warranted to clarify the magnitude and independence of these effects after adjusting for disease severity and treatment modality. Multivariable regression analyses are shown in Table 3.

## 4. Discussion

### 4.1. Key Findings

In this single-centre cohort of 174 oncology inpatients, we observed a substantial burden of anxiety and depressive morbidity: nearly half screened in the definite range on HADS, and approximately 40% received a clinician-documented psychiatric diagnosis during treatment. These figures sit at the upper end of contemporary oncology estimates and align with guideline summaries and recent syntheses indicating that anxiety and depressive disorders are common across cancer populations (often ≥20–30% over 12 months), with higher rates expected in hospital settings. Our findings, therefore, reinforce existing recommendations for routine case-finding and stepped care in oncology [2,4].

Psychological morbidity was not evenly distributed by tumour site. Patients with urogenital malignancies—especially those with cervical cancer—showed the most pronounced distress, with near-universal mixed anxiety–depressive presentations in our sample; bladder and prostate cancers also exhibited high rates, though less uniformly. These gradients are consistent with recent reports in gynaecologic oncology showing high point prevalences of anxiety and depression in cervical cancer (often >40–60%) and a sustained need for psychosocial support in this population [22,23].

Functional status emerged as a robust correlate: worse ECOG performance was independently associated with greater depressive symptom severity and higher odds of clinically significant distress in our multivariable models. This aligns with prospective and cross-sectional evidence linking poorer ECOG grades to higher anxiety/depression burdens and supports using ECOG as a pragmatic flag to trigger psycho-oncology assessment alongside symptom management [15].

By contrast, demographic contrasts were modest after adjustment. We did not find consistent differences by age group or residence (urban vs. rural), suggesting that, within an inpatient tertiary context, clinical status may overshadow sociodemographic influences. This pattern is compatible with recent inpatient HADS studies and reviews, noting heterogeneous or context-dependent associations of age and other demographics with distress [24,25].

To aid interpretation for non-specialists and in line with our aims, we briefly map what the HADS subscales capture. The HADS-A subscale indexes cognitive–affective anxiety (e.g., persistent worry, tension, restlessness, anticipatory fear), while the HADS-D subscale focuses on anhedonia/low positive affect (e.g., reduced interest and enjoyment, diminished outlook, slowed initiative), deliberately minimising somatic content that could be confounded by cancer- or treatment-related symptoms. In our context, this distinction is clinically informative:

(i) The stronger association of functional impairment (ECOG) with depressive severity is consistent with the HADS-D emphasis on loss of interest/drive in the face of fatigue, disability, or symptom burden.
(ii) The urogenital pattern (especially cervical cancer) plausibly reflects heightened HADS-A content (worry, tension around stigma, sexuality, fertility, continence) together with HADS-D features (anhedonia/withdrawal), aligning with our tumour site rationale.

Taken together, these domain signals help explain why HADS operates as a pragmatic proxy for clinically significant distress in oncology and why ECOG-informed, site-sensitive triage is warranted.

Taken together, these domain signals help explain why HADS operates as a pragmatic proxy for clinically significant distress in oncology and why ECOG-informed, site-sensitive triage is warranted [26,27,28,29,30]

### 4.2. Comparison with Literature

Our prevalence estimates align with—and are slightly above—the upper range reported in recent syntheses of psychiatric morbidity in oncology. Using diagnostic interviews or validated screens, large meta-analyses and guidelines typically place the 12-month prevalence of any mood/anxiety disorder or clinically relevant distress in the ~30–40% range across mixed cancer populations; in hospital settings, the rates tend to be higher, which likely explains our ~45% rate of definite anxiety/depression and ~40% with a clinician-documented diagnosis. Mitchell’s landmark meta-analysis across ~10,000 patients reported 38% prevalence of any mood/anxiety disorder, broadly comparable to our figure, while the 2023 ESMO guideline synthesises contemporary evidence that anxiety and depressive disorders are common and often under-recognised in routine care. A recent global review likewise cites ~39% 12-month prevalence of mental disorders among patients with cancer, further situating our inpatient cohort toward the upper end of expectations [2,4,31].

The tumour site gradient we observed—with the heaviest psychological burden in urogenital cancers, particularly cervical cancer—is consonant with prior work. Mehnert and colleagues reported that patients with female genital cancers experience some of the highest distress levels among tumour groups, a pattern reiterated in subsequent inpatient cohorts and survivorship studies; these data provide a plausible explanatory framework for our near-universal mixed anxiety–depressive presentations in the cervical subgroup, given the salience of sexuality, fertility, and body image concerns in this population [32,33,34].

By contrast, our head and neck subgroup showed only moderate distress, which diverges from several reviews highlighting a high psychosocial burden driven by treatment-related disfigurement, functional impairments (speech, swallowing), and stigma. Such discrepancies could reflect sample composition (older, predominantly male patients), under-reporting, or effective local supportive care pathways in our centre; nonetheless, the external literature underscores that head and neck cancer commonly carries substantial distress risk [35,36].

We also noted a gender trend (women > men) that attenuated after adjustment for cancer site; this is consistent with recent meta-analytic evidence of sex differences in anxiety/depression among patients with cancer and with guidance to maintain high sensitivity to under-detection in men, who may express distress differently or be less inclined to accept psychological help [37].

Finally, the clinical significance of distress suggested by our data signals toward longer length of stay, treatment disruption, and higher costs—mirroring external evidence linking anxiety/depression with greater healthcare utilisation and expenditures, as well as with poorer oncologic outcomes in some settings. A systematic review documents higher healthcare use and costs in distressed patients, and a large claims-based analysis shows significantly higher annual costs in cancer patients with anxiety/depression; meta-analytic work also associates depression/anxiety with increased cancer-specific and all-cause mortality, underscoring the importance of early identification and intervention [38,39,40].

Overall, our findings are therefore directionally consistent with the contemporary literature: high but context-dependent prevalence of anxiety/depression, especially pronounced in gynaecologic/urogenital malignancies; a recognised burden in head and neck cancer in most series; sex-related differences modulated by tumour type; and tangible implications for clinical outcomes and resource use that justify routine screening and integrated psycho-oncologic care.

### 4.3. Strengths and Limitations

**Strengths.** This analysis integrates objective clinical indicators (e.g., ECOG performance status, length of stay) with a standardised psychological measure (HADS) across a relatively sizeable, single-centre inpatient cohort (N = 174). Using HADS is advantageous in routine oncology because it is brief, widely adopted, and psychometrically supported, with well-established subscale cut-offs (≥8 “borderline/possible”, ≥11 “definite”) that facilitate consistent case-finding and comparability across studies [12,41,42]. The monocentric setting enhanced procedural consistency (the same psycho-oncology team, uniform charting and thresholds), reducing inter-observer variability. Moreover, reliance on real-world hospital records—rather than survey-only data—allowed us to couple symptom screening with clinician diagnoses and concrete utilisation outcomes (LOS, direct costs), increasing ecological validity and alignment with contemporary guidance that emphasises routine screening and integrated management of anxiety/depression in cancer care [2].

**Limitations.** As a retrospective, single-centre study, several sources of bias and imprecision must be acknowledged in line with STROBE guidance on observational research. Selection bias is plausible: an inpatient case-mix (and a radiotherapy-heavy service) likely over-represents more severe disease, inflating distress prevalence relative to ambulatory clinics; indeed, multiple studies report higher psychological distress in inpatients than outpatients [33,43,44]. Psychiatric assessment combined HADS screening with clinician judgement rather than a structured diagnostic interview for every patient; while appropriate for case-finding, HADS is a screening tool—not a diagnostic instrument—and may misclassify some cases even at standard thresholds [41]. Power was limited for several subgroup and outcome analyses (e.g., treatment interruptions), increasing uncertainty around those estimates. We also lacked granular staging/prognostic data and detailed treatment intent/toxicity variables, which can confound associations between distress and outcomes; disease severity, pain, and treatment-related toxicity are recognised determinants of anxiety and depression in oncology and should be measured explicitly in future work [2]. Finally, our snapshot of patients during a single hospitalisation precludes causal inference regarding whether psychological morbidity drives worse clinical trajectories or is a consequence of them; such cross-sectional limitations and potential residual confounding are inherent to retrospective designs and are highlighted in methodological guidance for observational studies [43].

### 4.4. Clinical Implications

Routine, protocolised screening for psychological distress should be embedded in day-to-day oncology—particularly in inpatient and advanced-disease settings—using validated tools (e.g., HADS, Distress Thermometer) with clear referral thresholds to psycho-oncology. This is consistent with contemporary guidelines and accreditation standards that call for systematic case-finding and stepped care, and with the ESMO Clinical Practice Guideline on anxiety and depression in adults with cancer. Implementing a standardised screen-and-refer pathway (initial screen at admission; re-screen before major treatment milestones; automatic referral for positive screens) is therefore justified by our data and by current guidance [2,45].

Given the extraordinary burden of distress in cervical cancer (and high rates in bladder/prostate), programmes should include early counselling and sexual health rehabilitation as part of routine care. Recent cross-guideline syntheses (ASCO, NCCN, ESO-ESMO, SOGC) emphasise structured management of sexual dysfunction (first-line non-hormonal approaches, with escalation as appropriate) and recommend multidisciplinary models that explicitly address psychosocial and relationship concerns—elements highly pertinent to gynaecologic oncology. Embedding these services (psycho-sexual counselling, pelvic floor/rehabilitation clinics, peer support) from diagnosis onward is likely to improve patient-reported outcomes and engagement with treatment [46].

For prostate cancer, vigilance for mood change during androgen-deprivation therapy (ADT) is warranted. A recent meta-analysis confirms a statistically significant increase in depression risk with ADT, supporting proactive monitoring (e.g., baseline and on-treatment mood screening), anticipatory guidance, and swift referral for evidence-based interventions when indicated (psychotherapy, pharmacotherapy) [9].

Because poor ECOG status strongly covaries with distress, oncology teams should co-manage frail patients early with palliative care and psycho-oncology to address symptom burden, coping, and decision support. Updated ASCO guidance recommends early palliative care involvement for patients with advanced cancer and significant symptom or quality of life concerns; integrating this alongside psycho-oncology is a pragmatic way to break the feedback loop between uncontrolled symptoms and escalating distress [47].

Finally, clinicians should treat depression-related non-adherence as a modifiable risk. Our signal toward more interruptions among severely depressed patients mirrors external evidence that mental health comorbidity is linked to lower receipt of guideline-concordant therapy and higher healthcare costs. Multidisciplinary tumour boards can operationalise this by adding a brief psycho-oncology summary to each case (screen result, current supports, adherence risks) and by triggering targeted interventions before dose omissions or treatment breaks occur [48,49].

## 5. Conclusions

This single-centre analysis indicates that psychiatric morbidity is highly prevalent among oncology inpatients, with especially elevated rates in urogenital malignancies—most notably cervical and bladder cancer. Across the cohort, nearly half screened in the definite range for anxiety and/or depression, and approximately two in five received a clinician-documented psychiatric diagnosis. Poor functional status (higher ECOG) was a robust correlate of distress, and female sex showed a higher burden in stratified analyses, underscoring the need to identify vulnerable subgroups early. We also observed trend-level signals linking severe depressive symptoms to longer hospital stays, treatment interruptions, and higher direct costs, suggesting clinically meaningful impacts on care delivery and resource use.

These findings support routine integration of mental health screening and stepped psycho-oncological care into standard oncology pathways: systematic case-finding with validated tools (operationalising distress via HADS thresholds), rapid referral to liaison psychiatry, and tailored interventions for high-risk groups (e.g., early psycho-sexual counselling in cervical cancer; mood monitoring during androgen-deprivation therapy in prostate cancer). Embedding such practices can improve patient-reported outcomes and may enhance treatment adherence and efficiency of care. While the retrospective, inpatient design limits causal inference, the consistency and magnitude of the observed burden justify institutional protocols that couple psychosocial assessment with multidisciplinary management.

Finally, given the single-centre, liaison psychiatry-assessed inpatient design and the modest sample size, generalisability is limited. Our conclusions should therefore be interpreted as exploratory and hypothesis-generating, and future prospective, multi-centre studies incorporating staging, treatment intent, and standardised adherence endpoints are warranted to validate these findings and quantify downstream effects on continuity of oncologic therapy, length of stay, and costs.

## Figures and Tables

**Table 1:** Baseline characteristics by cancer type.

Cancer Type	N (%)	Age, Median (Range)	Sex M/F	Residence U/R	ECOG 1/2/3/4	LOS Median (Range), Days	LOS Mean (SD), Days
All Patients	174 (100%)	68 (40–91)	85/89	134/40	18/56/95/5	10 (0–56)	
Lung	11 (6.32%)	77 (56–83)	7/4	6/5	2/0/9/0	6 (2–56)	12.73 (15.64)
Head and Neck (ORL)	42 (24.14%)	65.5 (49–91)	38/4	40/2	1/12/26/3	10.5 (0–50)	13.81 (13.31)
Breast	30 (17.24%)	67 (45–89)	0/30	23/7	8/8/13/1	6.5 (0–25)	7.67 (7.57)
Cervical	40 (22.99%)	63 (40–84)	0/40	34/6	4/19/17/0	10 (1–50)	12.7 (10.45)
Prostate	25 (14.37%)	73 (64–86)	25/0	15/10	3/7/14/1	6 (0–28)	9.24 (8.75)
Bladder	8 (4.60%)	77 (60–86)	4/4	3/5	0/3/5/0	13.5 (6–23)	13.88 (6.009)
Rectal	18 (10.34%)	71 (63–89)	11/7	13/5	0/7/11/0	6 (0–40)	8.94 (10.27)

Values are counts unless otherwise specified. Percentages for the “N (%)” column are within the total cohort (N = 174). ECOG = Eastern Cooperative Oncology Group performance status. U/R = urban/rural. LOS = length of stay.

**Table 2:** Proportion of anxiety/depression among psychiatry-assessed inpatients by tumour type.

Cancer Type (N)	HADS-A ≥ 8, n/N (%; 95% CI)	HADS-D ≥ 8, n/N (%; 95% CI)	Any Psych. Diagnosis, n/N (%; 95% CI)
Lung (n = 11)	5/11 (45.5%; 16.7–76.6)	5/11 (45.5%; 16.7–76.6)	5/11 (45.5%; 16.7–76.6)
Head and Neck (n = 42)	14/42 (33.3%; 19.6–49.5)	15/42 (35.7%; 21.6–52.0)	9/42 (21.4%; 10.3–36.8)
Breast (n = 30)	14/30 (46.7%; 28.3–65.7)	15/30 (50.0%; 31.3–68.7)	9/30 (30.0%; 14.7–49.4)
Cervical (n = 40)	38/40 (95.0%; 83.1–99.4)	37/40 (92.5%; 79.6–98.4)	22/40 (55.0%; 38.5–70.7)
Prostate (n = 25)	15/25 (60.0%; 38.7–78.9)	18/25 (72.0%; 50.6–87.9)	12/25 (48.0%; 27.8–68.7)
Bladder (n = 8)	8/8 (100.0%; 63.1–100.0)	8/8 (100.0%; 63.1–100.0)	6/8 (75.0%; 34.9–96.8)
Rectal (n = 18)	8/18 (44.4%; 21.5–69.2)	9/18 (50.0%; 26.0–74.0)	7/18 (38.9%; 17.3–64.3)
**Urogenital total ^†^ (n = 73)**	**61/73 (83.6%; 73.0–91.2)**	**63/73 (86.3%; 76.2–93.2)**	**40/73 (54.8%; 42.7–66.5)**
**Non-urogenital total ^‡^ (n = 101)**	**41/101 (40.6%; 30.9–50.8)**	**44/101 (43.6%; 33.7–53.8)**	**30/101 (29.7%; 21.0–39.6)**
**Overall (N = 174)**	**102/174 (58.6%; 50.9–66.0)**	**107/174 (61.5%; 53.8–68.8)**	**70/174 (40.2%; 32.9–47.9)**

^†^ Urogenital: cervical, prostate, bladder. ^‡^ Non-urogenital: lung, head and neck, breast, rectal. Exact *p*-values and 95% confidence intervals are reported for all proportions. For small subgroups (e.g., bladder, n = 8), binomial exact confidence intervals were used, and results should be interpreted as descriptive and exploratory. Group comparisons (urogenital vs. non-urogenital) used χ^2^ tests without correction for multiple comparisons (*p* < 0.01 for all outcomes).

**Table 3:** Multivariable regression analyses for depressive symptom severity and resource use outcomes.

**Panel A.** Predictors of severe depressive symptoms (logistic regression)		
**Predictor**	**OR (95% CI)**	***p*** **-value**
ECOG (per unit)	3.60 (2.09–6.19)	<0.001
Age (per year)	0.98 (0.93–1.03)	0.322
Female (vs. male)	0.56 (0.28–1.12)	0.104
Urban (vs. rural)	1.27 (0.42–3.84)	0.674
Urogenital cancer (vs. others)	1.44 (0.72–2.89)	0.308
**Panel B**. Determinants of hospital length of stay (LOS, days)—Linear regression (OLS)		
**Predictor**	**β (95% CI)**	***p*** **-value**
HADS-D (per point)	−0.10 (−0.86 to 0.66)	0.794
ECOG (per unit)	2.86 (−0.64 to 6.36)	0.108
Age (per year)	−0.33 (−0.62 to −0.04)	0.025
Female (vs. male)	1.05 (−3.66 to 5.76)	0.659
Urban (vs. rural)	−4.28 (−10.99 to 2.43)	0.209
Urogenital cancer (vs. others)	−0.71 (−5.33 to 3.90)	0.760
**Panel C**. Determinants of direct treatment cost—Linear regression on log-cost		
**Predictor**	**%Δ cost per unit (95% CI)**	***p*** **-value**
HADS-D (per point)	+1.5% (−9.3% to +13.5%)	0.799
ECOG (per unit)	+104.2% (+21.0% to +244.8%)	0.008
Age (per year)	−5.2% (−9.0% to −1.2%)	0.011
Female (vs. male)	+40.7% (−28.4% to +176.5%)	0.319
Urban (vs. rural)	−64.8% (−86.5% to −8.2%)	0.033
Urogenital cancer (vs. others)	−22.8% (−60.3% to +50.1%)	0.442

ECOG = Eastern Cooperative Oncology Group performance status (1 = fully active; 4 = bedbound). Urogenital cancers: cervical, prostate, bladder; non-urogenital: lung, head and neck, breast, rectal. Direct cost (‘Cheltuieli Directe’) is recorded in RON; log-cost model excludes zero-cost stays. Model sizes: Panel A: N = 173 (McFadden pseudo-R^2^ = 0.165; LLR *p* = 1.75 × 10^−7^); Panel B: N = 136 (R^2^ = 0.066; model *p* = 0.174); Panel C: N = 127 (R^2^ = 0.142; model *p* = 0.0049).

## Data Availability

The original contributions presented in this study are included in the article. Further inquiries can be directed to the corresponding authors.

## Abbreviations

The following abbreviations are used in this manuscript:

ANOVA	Analysis of Variance
CI	Confidence Interval
HADS	Hospital Anxiety and Depression Scale
HADS-A	Hospital Anxiety and Depression Scale, Anxiety Subscale
HADS-D	Hospital Anxiety and Depression Scale, Depression Subscale
ICD-10	International Classification of Diseases, 10th Revision
LOS	Length of Stay
OR	Odds Ratio
SD	Standard Deviation
